# deepBBQ: A Deep Learning Approach to the Protein Backbone Reconstruction

**DOI:** 10.3390/biom14111448

**Published:** 2024-11-14

**Authors:** Justyna D. Kryś, Maksymilian Głowacki, Piotr Śmieja, Dominik Gront

**Affiliations:** Faculty of Chemistry, University of Warsaw, Pasteura 1, 02-093 Warsaw, Poland; jkrys@chem.uw.edu.pl (J.D.K.); m.glowacki10@student.uw.edu.pl (M.G.); p.smieja2@uw.edu.pl (P.Ś.)

**Keywords:** atomic reconstruction, structural bioinformatics, deep learning, coarse-grained modeling, convolutional neural network

## Abstract

Coarse-grained models have provided researchers with greatly improved computational efficiency in modeling structures and dynamics of biomacromolecules, but, to be practically useful, they need fast and accurate conversion methods back to the all-atom representation. Reconstruction of atomic details may also be required in the case of some experimental methods, like electron microscopy, which may provide Cα-only structures. In this contribution, we present a new method for recovery of all backbone atom positions from just the Cα coordinates. Our approach, called deepBBQ, uses a deep convolutional neural network to predict a single internal coordinate per peptide plate, based on Cα trace geometric features, and then proceeds to recalculate the cartesian coordinates based on the assumption that the peptide plate atoms lie in the same plane. Extensive comparison with similar programs shows that our solution is accurate and cost-efficient. The deepBBQ program is available as part of the open-source bioinformatics toolkit Bioshell and is free for download and the documentation is available online.

## 1. Introduction

With the concept of coarse-grained molecular modeling introduced in the 1970s [1], molecular simulations became significantly more efficient. With this achievement, however, the need to recover all-atom structure arose. Since the 1980s [2], multiple approaches have been proposed to the problem of recalculating atomic details from a coarse grained representation of a protein conformation, typically from just the Cα positions. This task is generally solved in two steps. Firstly, the Cartesian coordinates of all backbone atoms are calculated. Then, amino acid side chains are reconstructed based on the backbone conformation.

For the past few decades, many algorithms have been devised to solve the backbone reconstruction problem. Among these approaches, one can find analytical solutions [3], Dead End Elimination algorithm [4], Dynamic Programming method [5], Deep Machine Learning [6], energy minimisation [2,7,8], Gaussian Mixture Models [9] and prediction of Φ, Ψ dihedral angles [10]. In some methods, reconstruction process is followed by energy optimization to improve the result. A distinct category of algorithms utilize fragment libraries [11,12,13,14] derived from known structures to locate possible structures that do not violate a specified Cα trace. The most favorable fragments to construct the entire backbone are selected using energy-based [15], homology-based [16], or geometric [5,17,18] criteria, or a combination of them [19]. Another prevalent approach relies on an observation that the local internal geometry of a Cα trace typically determines the location of the remaining atoms of a protein backbone. This is especially true for regular conformations: helices and sheets, which are stabilised by a network of hydrogen bonds. In this approach, first introduced by Milik et al. [20], internal geometry of four Cα atoms of a tetrapeptide is uniquely described by three degrees of freedom; traditionally $r_{i,i+2}$, $r_{i+1,i+3}$ and $r_{i,i+3}$ distances are utilised. A three-dimensional grid is constructed based on these $r_{i,i+2}$, $r_{i+1,i+3}$ and $r_{i,i+3}$ internal distances. Therefore, each element of that grid aggregates tetrapeptides that are structurally similar to each other. Local Cartesian coordinates of N, C and O backbone atoms are calculated in a Local Coordinate System (LCS) and averaged separately for each element of the grid. The main advantage of this method is the simplicity of its application. To reconstruct a protein backbone, one has to calculate $r_{i,i+2}$, $r_{i+1,i+3}$ and $r_{i,i+3}$ internal distances and then retrieve local N, C and O Cartesian coordinates from the correct bin of the grid. In the last step, these local coordinates are transformed to the global coordinate system. Due to the ease of implementation and computational efficiency, the Milik’s approach has been prevalent in the field and implemented by popular backbone reconstruction methods such as BBQ (Backbone Building from Quadrilaterals) [21], Pulchra [22] and REMO [23].

**Figure 1:**
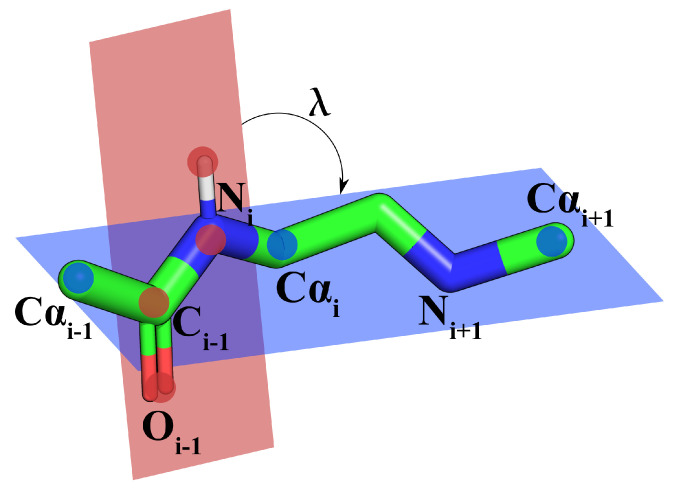
λ angle parameterizing the backbone conformation.

Since the original publication of the BBQ software, it has been extensively used in various modeling scenarios. The experience we gained over these years has shown that the significant weakness of the algorithm results directly from its design. Besides alpha carbons (given as the program input), there are three other heavy backbone atoms: carbonyl carbon, carbonyl oxygen and amide nitrogen. The reconstruction process assumes their three Cartesian coordinates are independent variables and thus treats the problem as a 9-dimensional. However, this assumption is incorrect and may result in stereochemical errors such as incorrect bond lengths and planar angles far from their equilibrium values. In addition, the BBQ method makes incorrect predictions for conformations rarely observed in the PDB, such as loops. This contribution presents a novel protein backbone reconstruction method that alleviates most of these problems. We assume that the peptide plate atoms (C*_i_*, O*_i_*, N_*i*+1_ and optionally the amide hydrogen of the (i+1)-th residue) do, in fact, always lay exactly on the same plane; in other words: that the ω dihedral angle assumes either −180 or 180 degrees. Under such an assumption, the 9-dimensional problem can be reduced to just one dimension. In the new approach, the only degree of freedom that has to be established for each amino acid residue is the dihedral angle between a peptide plate and a reference plane. In this work, we follow the convention by Purisima et al. [2], where a $\lambda_{i}$ dihedral angle is defined for an *i*-*th* residue as an angle between the two planes: the peptide plate between C$\alpha^{i-1}$ and C$\alpha^{i}$, and the plane defined by C$\alpha^{i-1}$, C$\alpha^{i}$, C$\alpha^{i+1}$ (shown in the Figure 1).

Knowing the Cα positions and the λ angle values, Cartesian coordinates of all backbone atoms can be easily recovered, making λ prediction a viable candidate for a backbone reconstruction method. In this contribution, which stems from our previous BBQ method, we engineered a deep neural network to predict these λ values; hence the name of the new program: deepBBQ.

The overview of this manuscript is as follows: the Section 2 describes the algorithm in detail. It discusses the architecture, training, and validation of the ML model we used in this study. The convolutional neural network we devised takes several geometric features computed solely from Cα positions as well as an amino acid sequence and predicts the λ angle as introduced above. The following Section 3 provides a thorough test of the method and a comparison with existing approaches conducted on standard benchmark sets used in the field. To avoid a situation where the protein in the test set is a homolog of an element in the training set, we also provided a test on de novo designed proteins. These tests prove the deepBBQ method to be superior over all traditional (i.e., not ML-based) algorithms. Finally, in the Section 4 we summarise our findings as well as provide prospects for future development of backbone reconstruction algorithms.

## 2. Methods

### 2.1. Protein Backbone Reconstruction

The deepBBQ reconstruction algorithm is quite simple. A peptide plate of ideal planar geometry is transformed from a local to the global coordinate system so its C$\alpha^{1}$ − C$\alpha^{2}$ vector overlays C$\alpha^{i}$ − C$\alpha^{i+1}$ axis. Then, the peptide plate is rotated around the C$\alpha^{i}$ − C$\alpha^{i+1}$ axis by $\lambda_{i}$ dihedral angle; the angle itself is predicted by a deep neural network based on Cα trace geometry. The method uses idealised alanine geometry to reconstruct a peptide plate of any amino acid type except proline, where an idealised proline peptide plate is employed. Moreover, alanine and proline peptide plates may be in cis or trans conformation. Below, we describe the λ prediction network architecture and training. Validation and comparison between deepBBQ and other methods are given in the Section 3.

### 2.2. deepBBQ Neural Network

We devised a deep convolutional neural network to obtain $\lambda_{i}$ values. The prediction is based on 37 values per residue as input. For a given residue *i*, the input features are:

(a) 21 binary values for one-hot-encoded residue type representation, corresponding to 20 canonical amino acids and the ’X’ symbol representing unknown residue types
(b) 6 floating point values corresponding to distances between C$\alpha^{i}$ and C$\alpha^{i\pm j}$ for j∈{3,4,5}
(c) 4 integer values for the number of Cα atoms present within 4, 4.5, 5 and 6 Å from C$\alpha^{i}$
(d) 3 binary values for one-hot-encoded secondary structure information (either helix, sheet or loop)
(e) One binary value as a flag for the cis/trans classification of the peptide bond between amino acids *i* and (i+1)
(f) 2 integer values corresponding to the number of hydrogen bonds involved in helices and strands

All these features are obtained from input Cα atom coordinates and have been used for a past few decades to represent a protein structure at a Coarse-Grained level [24]. Local distances along a Cα trace (b) have been traditionally employed to distinguish between compact (helical-like) and extended local conformations. The number of spatial neighbors (c) indicates regions of dense packing, where backbone conformations are more rigid. The HECA [25] (H-E-C Assigner) method assigns the secondary structure classification (d) for each amino acid residue. The number of hydrogen bonds (f) is computed for each Cα atom according to the Coarse-Grained H-bond potential [26]. Due to its mean-field design, this scoring function detects H-bonds only in regular secondary structure elements. Here we believe this feature allows the network to differentiate between extended loops and strand conformations, which are hard to distinguish based on any other feature we use. Finally, a cis/trans classification of a peptide bond (e) is based on the C$\alpha^{i}$−C$\alpha^{i+1}$ distance: pseudo-bonds shorter than 3.5 Å are classified as cis [27,28].

**Figure 2:**
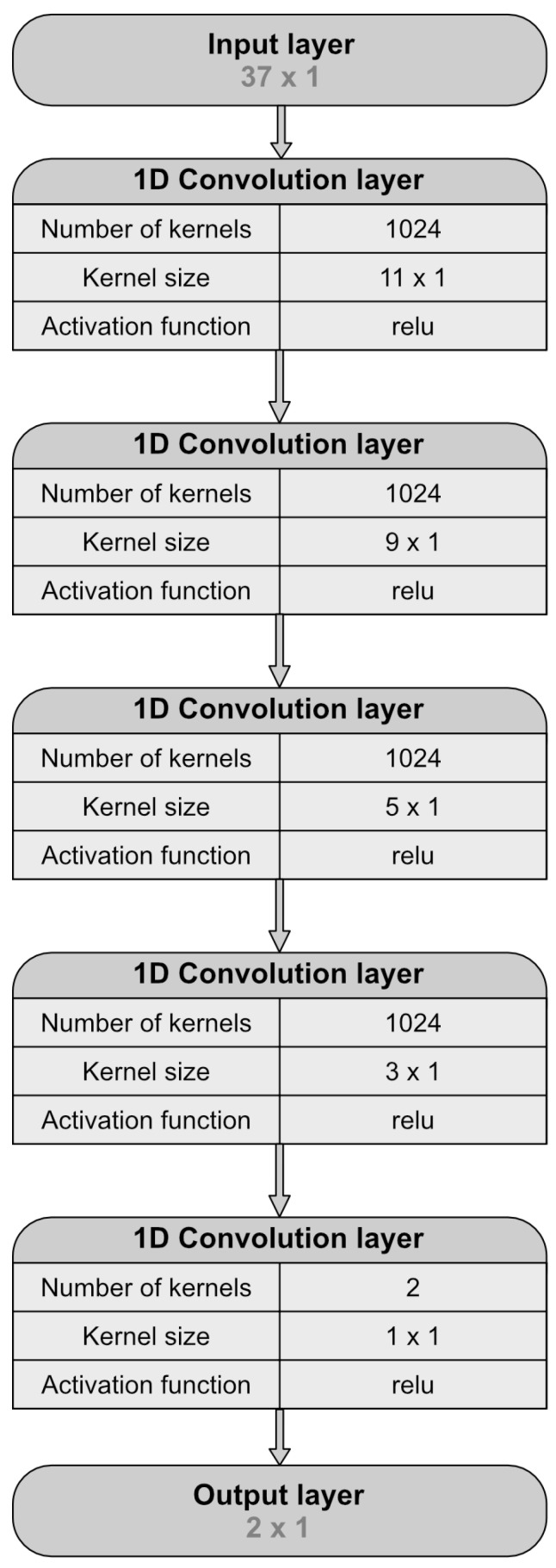
Architecture of the deepBBQ neural network.

To reconstruct the backbone of a protein structure of *K* residues, the user must provide Cartesian coordinates of *K* Cα atoms. Based on this input the deepBBQ program calculates a matrix of 37×K input features. The neural network consists of five one-dimensional convolutional layers connected sequentially. The first four layers consist of 1024 kernels of sizes 11, 9, 5 and 3 and the last layer consists of two kernels of size one, providing the two output values corresponding to the sine and cosine of the λ angle of the given residue. To train the network, we used the mean squared error loss function based on the two values. While a single parameter λ per residue is required for coordinate reconstruction, the periodic nature of a dihedral angle imposes considerable difficulties in devising a loss function. To remedy this, our neural network predicts values of sin(λ) and cos(λ) instead and the value of λ in the range of [−π,π] is then calculated as $\arctan\left(\frac{\sin\lambda }{\cos\lambda }\right)$. The network architecture is depicted in the Figure 2.

### 2.3. Training Data and Tools

We used a non-redundant protein structure dataset provided by the PISCES server [29,30] with sequences culled at 40% identity and resolution of 1.6 Å, containing 6695 proteins. The dataset was filtered using Bioshell [31] software. We removed proteins with incomplete or incorrect fragments, e.g., with missing residues, missing backbone atoms, or important stereochemical errors. After the filtering, 6396 chains remained and were used to train the network. The training was performed in Python using tensorflow library [32]. Finally, the method has been implemented as part of the BioShell [31] suite in C++ with frugally-deep [33], a simple header-only library, providing an interface to the neural network.

### 2.4. Testing Set

The set of protein structures used for testing was compiled by selecting one remote homolog for each structure of the training set. We used the Jackhmmer [34] program of the HMMER package to search through protein sequences from the PDB database [35] using each amino acid sequence from the training set as a query. We attempted to select a sequence with an e-value close to 10^−7^ for each query. This was not possible for every query since some of PDB deposits have no homologous structures in this database, or the protein sequences are too similar to one another (e.g., point mutants). The e-value range we assumed as a selection criterion was manually adjusted to provide us with remote but still homologous hits, typically at the edge of detectability by sequence identity (below 40%). We subsequently filtered this set by removing close homologs that somehow entered the test set and structures with missing residues, alternative locations and other structural errors, as detected by the BioShell package. Finally we obtained a test set comprising 2882 protein chains. The complete list is provided in Supplementary Materials. To benchmark the new method presented in this study, we isolated Cα coordinates from PDB files and ran deepBBQ on such input files.

### 2.5. De Novo Testing Set

To ensure our tests haven’t been biased by homolog contamination, we decided to compile a de novo testing set. We collected all the de novo designed protein structures, that are currently available in the PDB database. We found around 1500 CIF files that have been classified as De Novo. However, many of these contain only a designed peptide bound to a natural protein. Therefore, we restricted the set by selecting only chains at least 30 amino acids long and chose deposits of resolution 2.5 Å or better. Subsequently, we removed any chain that was identical in 30% or more to any protein from the training set, which might have happened accidentally. Finally, we used the clust program of the BioShell package to cluster the set of de novo proteins with the distance between two given sequences defined as:

$$d\left(sequence_{1},sequence_{2}\right)=1-sequence\;identity\left(sequence_{1},sequence_{2}\right)$$

The program performed hierarchical agglomerative clustering with the single-linkage rule, producing 105 clusters selected at a 30% sequence identity level. This means that any protein sequence from a cluster is identical in less than 30% to any protein sequence that belongs to another cluster. The final set of proteins consists therefore of 105 sequences, one per cluster.

## 3. Results

**Figure 3:**
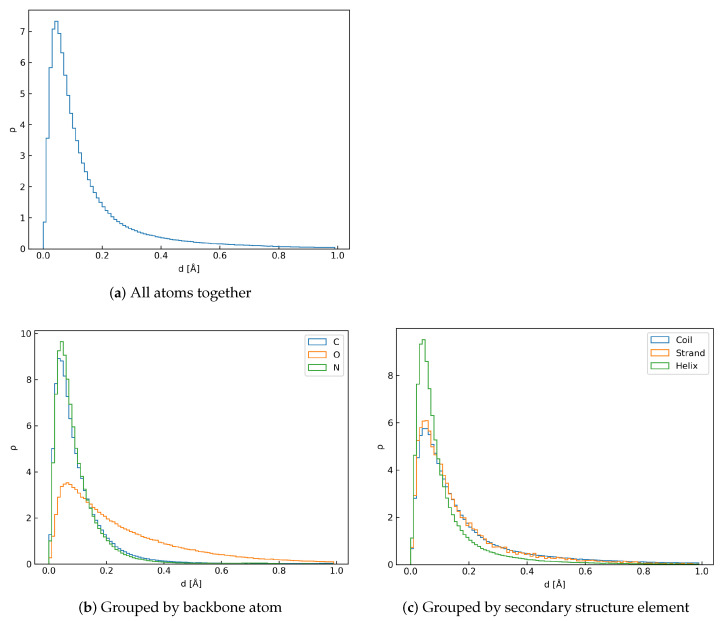
Density histograms of rmsd values [Å] between original backbone positions and ones reconstructed by deepBBQ for the test set. Histograms were cut off at 1.0 Å for readability.

**Figure 4:**
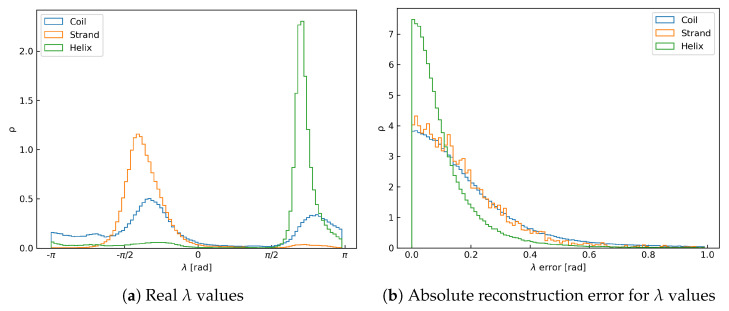
Density histograms of real λ values and λ reconstruction errors for the test set, grouped by secondary structure element. Accounting for λ periodicity, reconstruction error of λ is between 0 and π, but its histogram was cut off at 1.0 for readability.

**Figure 5:**
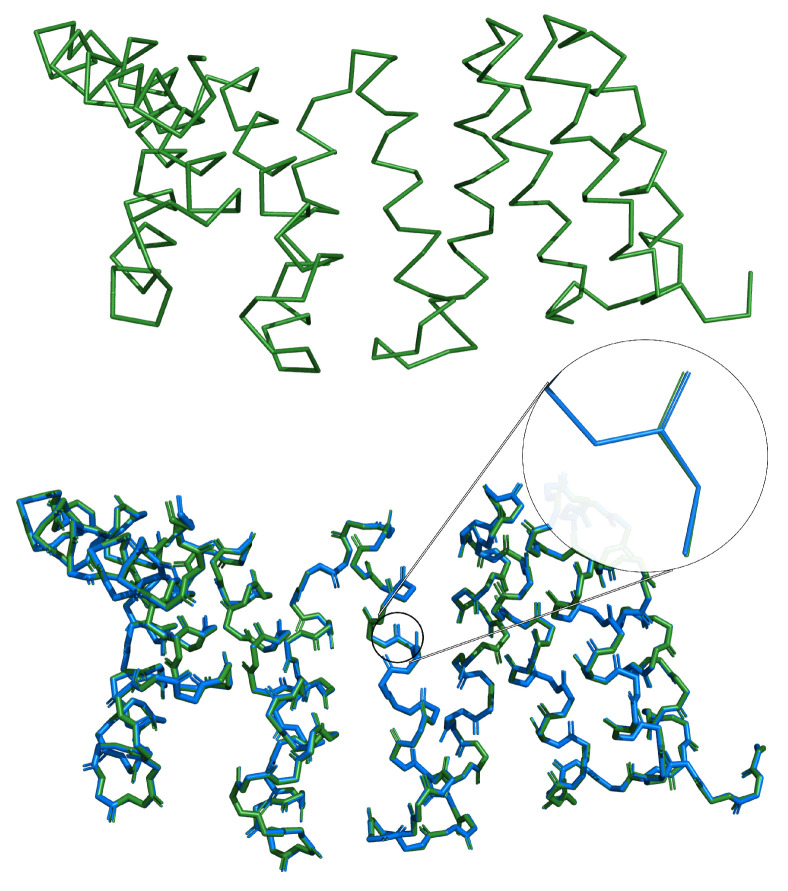
Example protein (4Y6W) rebuilt using deepBBQ. The rebuilt structure is shown in blue, while the superimposed native structure is in green.

The algorithm presented in this contribution was able to accurately and efficiently rebuild full-atom protein backbone conformations from respective Cα traces. The average reconstruction error, i.e., coordinate root mean square deviation (crmsd) value measured over all heavy backbone atoms for each test protein, was 0.19 ± 0.32 Å, and the most probable value (mode) was 0.045 Å. A histogram of these error values collected for the test set is shown in the Figure 3a. We have also investigated how the reconstruction error depends on the secondary structure type (see Figure 3c and Table 1). It is clear that the residues involved in helices perform visibly better than those in strands and loops. It can be attributed to the fact that the orientation of a peptide plate in helical conformations is very well-defined. This makes the prediction of respective λ values much easier for the network. As shown in Figure 4, both the distribution of λ and its reconstruction error are narrower for the helices than the other structure elements. Performance for β-strands is worse, explained by the larger flexibility of the extended secondary structure, which results in a broader peak on the respective histogram of λ values (c.f. Figure 4a). Unsurprisingly, the results are the worst for loops, which are the least organized. In their case, the λ distribution is bimodal since loops in proteins can adopt both helical-like and extended conformations. Figure 3b and Table 1 present the reconstruction error separately for each atom type. These results show that carbonyl oxygen atoms are the most prone to reconstruction errors. These atoms are the furthest away from the axis by each peptide plate is rotated. Therefore, any inaccuracies in predicted λ angles introduce the most significant error in the Cartesian space. Another important source of error is high mobility of residues, located near the chain termini and chain breaks. Overall, the deepBBQ algorithm outperforms most competing approaches (see Section 3.1). Thanks to the simple implementation in C++ it is also computationally efficient. An example of deepBBQ reconstruction of 4Y6W PDB deposit superimposed on the experimental structure is shown in Figure 5. The zoom-in in the figure depicts that backbone reconstructed for the helical conformation is nearly identical to the original one; loops and strand are, therefore, the main source of reconstruction inaccuracy.

### 3.1. Comparison with Other Methods

**Figure 6:**
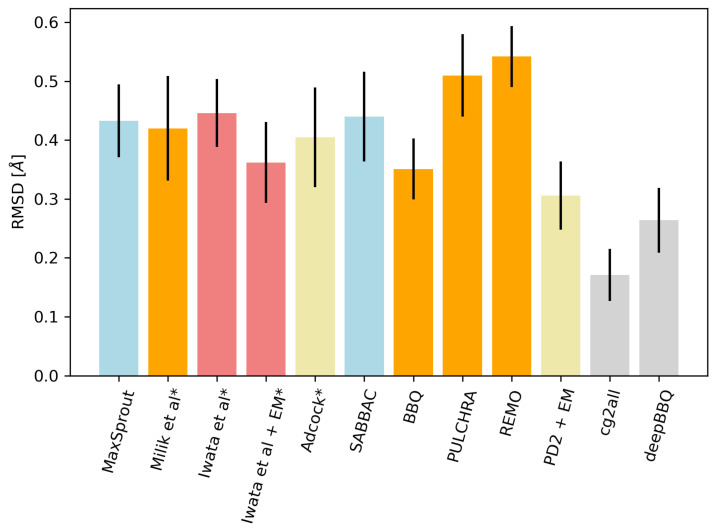
Comparison of backbone reconstruction accuracy for different methods. The bar heights represent RMSD between real and reconstructed backbone positions. The methods are listed chronologically, based on the time of their publication. Methods labeled “+ EM” include an additional energy minimization step. Blue bars indicate methods using PDB structure fragments, orange bars mean Milik method peptide plate insertion methods, red means methods using torsion angle prediction, gray is for machine learning methods and light yellow for others. Protein test sets come from Table 2 for all methods except for those labeled with “*” (Milik et al., Iwata et al. and Adcock), for which they were taken from their corresponding articles [4,10,20].

To compare deepBBQ with other approaches, we run the program on the test set from Moore et al. [9]. We decided to rely on this benchmark, because it provided the most exhaustive set of reconstruction tools we could find in the literature. Some of these methods are no longer available online, and repeating such a study would impose serious technical difficulties. Therefore, we simply run our calculations with deepBBQ and cg2all [6], which, according to the authors, is best performing algorithm. These resulted in two additional columns added to the Moore et al. [9] values; results are shown in Table 2 and Figure 6.

Indeed, deepBBQ outperforms most of the other methods; the only program that obtains better accuracy is cg2all, which, in addition to the backbone atoms, also rebuilds protein side chains. It uses, however, a much more complex machine learning model, which makes it significantly slower. While deepBBQ uses the frugally-deep library, which does not implement parallelism directly, one can run multiple program instances simultaneously for different input files. Running deepBBQ this way for the protein test set from the Table 2 took 10.31 s, while cg2all took 189.35 s (both calculations were ran on the same Intel Xeon E5649 CPU). Even when run on a single core, deepBBQ outperforms cg2all when it utilises the entire processor.

### 3.2. Reconstruction of De Novo Proteins

**Figure 7:**
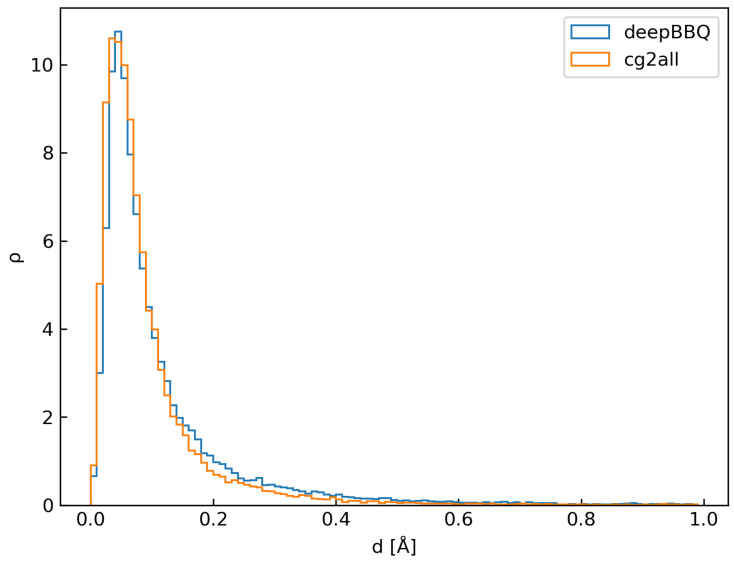
Reconstruction error measured for deepBBQ and cg2all methods on de novo testing set.

We devised a testing set comprising 105 de novo designed structures to further investigate the reconstruction accuracy. Such human-made proteins, by definition, have no homologs that may be found in the universe of life. Moreover, we ensured each test protein from this set was at most 30% identical to any protein from the training set. Results are shown in the Figure 7, which provides a histogram of reconstruction error in Å both for deepBBQ and cg2all methods.

To our surprise, deepBBQ performed significantly better in this test: mean reconstruction error was 0.14 ± 0.27 Å and the most probable value (mode) was 0.045 Å (values reported above for the first test set were 0.19 ± 0.32 Å and 0.045 Å, respectively). We explain this result by the fact, that de novo designed proteins are mostly α-helical. It is much easier to design an α bundle than a β barrel, which has resulted in the high popularity of α-bundles in the field of protein design. This, however, introduced a considerable bias to our de novo test.

### 3.3. Accuracy of the Two Steps of the Reconstruction Process

As described in the Section 2, the backbone reconstruction process consists of two stages: λ prediction and conversion to Cartesian coordinates, at both of which an error can be introduced. Errors in the first step appear because of the flaws of λ prediction, while errors in the second step stem primarily from the assumption that a peptide plate is flat and its bond angles equals to idealised values. To find out how much of a reconstruction error can be attributed to each of these two factors, we assumed λ values were predicted exactly (i.e., with no error). For this comparison, we used a set of 43 test proteins; for each of these, we calculated the real values of λ based on the actual experimental structure. Then we ran the backbone Cartesian coordinate reconstruction using these true (experimental) λ angles and calculated crmsd. For the comparison, we also reconstructed backbone atoms of each of the 43 test cases as described above, i.e., based on λ values predicted by the deepBBQ neural network. A comparison between these two sets of results is given in the Table 3. As expected, the reconstruction error for the true λ values is significantly lower, suggesting that the error is primarily caused by the imperfect λ prediction, rather than the geometry assumptions present in the second step.

## 4. Discussion

A few important conclusions can be drawn from the analysis of the results. Firstly, machine learning approaches, represented here by cg2all and deepBBQ, provide much better accuracy than the traditional solutions, such as BBQ, PULCHRA, and others, shown in the Figure 6. Secondly, one can obtain better results within the machine learning framework by increasing a network size and complexity. Indeed, the two ML-based approaches compared in this study differ significantly in the input features they utilize. Both methods rely on a local Cα trace geometry; cg2all however also includes explicit map of spatial neighbors, i.e., a contact map computed from Cα positions. This type of 2D information certainly gives cg2all an advantage over deepBBQ, which works solely on 1D protein structure representation. The two machine models also differ in their architecture. While deepBBQ utilizes a simple convolutional neural network, cg2all includes a graph neural network with attention layers. The higher accuracy, however, comes at the cost of increased computation time, as shown by the comparison described in this article. Finally, the reconstruction results obtained for the true λ values (Table 3) show, that the assumption about the planarity of a plate plate is quite realistic and does not introduce significant reconstruction errors on its own. The average prediction error of λ angles from the neural network presented in this study was 0.14 radians. However, the distribution of these values is quite broad, especially in loops and β-strands. We believe the accuracy of the deepBBQ approach can be further improved if λ values are predicted more accurately. Given the relatively long tail of the λ error distribution, this goal seems feasible.

In summary, deepBBQ provides a competitive solution for protein backbone coordinates reconstruction, ensuring high accuracy with good performance. It can be used as a part of the BioShell package, but it is also compiled as a standalone program with convenient command-line interface. The source code, as well as documentation, have been made publicly available.

## Figures and Tables

**Table 1:** Mean and mode of the distribution ρ of distance *d* between original backbone atoms and their reconstruction by deepBBQ (i.e., reconstruction error): (**left**) grouped by element and (**right**) grouped by Secondary Structure Element (SSE) type.

Atom	Mean [Å]	Mode [Å]		SSE	Mean [Å]	Mode [Å]
C	0.12 ± 0.18	0.045		Helix	0.12 ± 0.20	0.042
N	0.11 ± 0.15	0.039		Strand	0.18 ± 0.23	0.057
O	0.33 ± 0.46	0.069		Coil	0.23 ± 0.38	0.056

**Table 2:** Values of rmsd [Å] between the real backbone positions and reconstructions provided by the methods. Values for deepBBQ and cg2all were calculated by us, while all the others come from [9].

PDB Code	PD2 + Min	BBQ	MaxSprout	PULCHRA	SABBAC	REMO	deepBBQ	cg2all
4EO0	0.211	0.203	0.392	0.348	0.286	0.437	0.229	0.147
4F7H	0.293	0.356	0.505	0.471	0.480	0.551	0.318	0.232
4EXO	0.264	0.289	0.447	0.459	0.436	0.530	0.235	0.190
4F7V	0.444	0.410	0.436	0.617	0.450	0.643	0.377	0.199
4FAK	0.285	0.325	0.461	0.461	0.366	0.514	0.184	0.207
4ANN	0.238	0.292	0.405	0.452	0.445	0.514	0.320	0.221
4FFK	0.355	0.447	0.390	0.604	0.441	0.607	0.361	0.264
4FD5	0.318	0.319	0.426	0.421	0.406	0.515	0.253	0.131
4AVX	0.227	0.314	0.360	0.402	0.318	0.474	0.228	0.169
4EV1	0.239	0.304	0.396	0.411	0.385	0.504	0.219	0.159
4EG9	0.317	0.427	0.478	0.504	0.350	0.531	0.318	0.209
4EIU	0.334	0.339	0.468	0.587	0.555	0.556	0.282	0.205
4F78	0.364	0.424	0.552	0.558	0.477	0.616	0.317	0.253
4FCU	0.298	0.342	0.455	0.458	0.501	0.502	0.266	0.144
4FBR	0.404	0.399	0.528	0.586	0.552	0.622	0.256	0.149
4FB7	0.248	0.335	0.344	0.461	0.359	0.506	0.183	0.193
4FIK	0.374	0.356	0.288	0.568	0.575	0.563	0.326	0.134
4FAT	0.227	0.413	0.359	0.540	0.431	0.632	0.337	0.124
4FE3	0.275	0.318	0.489	0.449	0.450	0.507	0.207	0.119
4FCS	0.329	0.326	0.458	0.504	0.452	0.533	0.198	0.128
4E9L	0.267	0.315	0.403	0.546	0.544	0.540	0.229	0.118
4F8X	0.330	0.380	0.538	0.562	0.488	0.542	0.301	0.117
4FHG	0.318	0.350	0.397	0.549	0.425	0.506	0.298	0.126
4EYO	0.271	0.368	0.384	0.569	0.344	0.558	0.233	0.201
4F8J	0.279	0.354	0.388	0.510	0.370	0.458	0.235	0.214
3VTF	0.310	0.342	0.468	0.551	0.401	0.538	0.183	0.127
4FE9	0.392	0.406	0.433	0.573	0.534	0.568	0.254	0.152
4AVZ	0.352	0.369	0.480	0.568	0.489	0.602	0.244	0.145
mean	0.306	0.351	0.433	0.510	0.440	0.542	0.264	0.171
σ	0.058	0.052	0.062	0.070	0.076	0.052	0.055	0.044

**Table 3:** Comparison of results for deepBBQ method for real λ angle (the true column) and the one predicted by neural network (the predicted column).

PDB Code	Nres	True [Å]	Predicted [Å]
1CRN	46	0.051	0.615
6PTI	58	0.057	0.234
1CTF	68	0.165	0.344
1UBQ	76	0.055	0.432
2OZ9	104	0.043	0.143
4EO0	115	0.049	0.205
2MHR	118	0.165	0.422
4F7H	135	0.160	0.352
2FOX	138	0.032	0.355
5NLL	138	0.112	0.351
4EXO	144	0.127	0.300
4F7V	161	0.157	0.393
4FAK	163	0.115	0.205
2ALP	198	0.103	0.297
4ANN	210	0.149	0.317
4FFK	214	0.113	0.387
4FD5	216	0.110	0.252
4AVX	223	0.109	0.250
4EV1	229	0.062	0.260
4EG9	232	0.141	0.308
4EIU	241	0.134	0.299
4F78	254	0.127	0.323
4FCU	262	0.120	0.315
4FBR	273	0.061	0.291
4FB7	274	0.055	0.150
4FIK	278	0.125	0.339
2PRK	279	0.094	0.258
4FAT	280	0.146	0.339
4FE3	295	0.060	0.235
5CPA	307	0.172	0.391
4FCS	315	0.079	0.195
4E9L	318	0.085	0.234
3APP	323	0.113	0.301
4F8X	335	0.132	0.309
9WGA	340	0.316	0.367
4FHG	342	0.138	0.306
4EYO	358	0.088	0.225
4F8J	365	0.088	0.249
3VTF	432	0.068	0.205
2CTS	437	0.096	0.308
4FE9	450	0.097	0.250
1TIM	494	0.412	0.465
4AVZ	608	0.080	0.246
Avarage		0.115	0.303

## Data Availability

The source code is available at https://bitbucket.org/dgront/bioshell (accessed on 10 October 2024). The website https://bioshell.readthedocs.io (accessed on 10 October 2024) contains full reference documentation.

## Supplementary Materials

The following supporting information can be downloaded at: https://www.mdpi.com/article/10.3390/biom14111448/s1, S1.txt file provides the de novo test set, comprising de novo designed proteins, used in the final benchmark.
